# Drowning Among Children 1–4 Years of Age in California, 2017–2021

**DOI:** 10.5811/westjem.20356

**Published:** 2024-09-09

**Authors:** Phyllis F. Agran, Diane G. Winn, Soheil Saadat, Jaya R. Bhalla, Van Nguyen Greco, Nakia C. Best, Shahram Lotfipour

**Affiliations:** * University of California Irvine School of Medicine, Department of Pediatrics, Irvine, California; † American Academy of Pediatrics Orange County, Newport Beach, California; ‡ University of California Irvine School of Medicine, Department of Emergency Medicine, Irvine, California; § University of California Irvine, Sue and Bill Gross School of Nursing, Irvine, California

## Abstract

**Background and Objectives:**

Drowning, the leading cause of unintentional injury death among California children less than five years of age, averaged 49 annual fatalities for the years 2010–2021. The California Pool Safety Act aims to reduce fatalities by requiring safety measures around residential pools. This study was designed to analyze annual fatality rates and drowning incidents in California among children 1–4 years of age from 2017–2021.

**Methods:**

We identified fatalities, injury hospitalizations, and emergency department (ED) visits from California state vital statistics death data and state hospital and ED discharge data using the EpiCenter California Injury Data Online website.

**Results:**

Over the five-year study period, 4,166 drowning incidents were identified: 234 were fatalities, 846 were hospitalizations, and 3,086 were ED visits. The observed difference in fatality rates from 2017 to 2021 failed to achieve statistical significance (*P* = 0.88). Location-based analysis of the 234 fatal drowning incidents revealed that pools were the most common injury site, accounting for 65% of the cases.

**Conclusion:**

Drowning remains the leading cause of unintentional, injury-related death among California children 1–4 years of age, as the annual rate of fatality over the five-year study period did not decline. While the EpiCenter California Injury Data Online website is excellent for analyzing annual rates of drowning incidents among California residents over time, it is limited in providing insight into modifiable risk factors and event circumstances that can further inform prevention. The development of robust integrated fatal and non-fatal local, state, and national systematic data collection systems could aid in moving the needle in decreasing pool fatalities among young children.

Population Health Research Capsule What do we already know about this issue?

*Drowning is the leading cause of unintentional, injury-related death among children 1–4 years of age in the US and California.*

What was the research question?

*We examined recent fatality rates and characterized drowning incidents among California children 1–4-years of age.*

What was the major finding of the study?

*Annual drowning fatality rates (2017–2021) did not decline (*P* = 0.88), and 65% of fatalities occurred in pools.*

How does this improve population health?

*This study offers a new perspective on the need for robust data collection systems that aid in moving the needle in decreasing pool fatalities among young children.*

## INTRODUCTION

Drowning is the leading cause of unintentional, injury-related death among children 1–4 years of age in the US and California, and the second leading cause of unintentional, injury-related death among children 5–9 years of age.^1^ ^,^ ^2^ ^,^ ^3^ For the years 2010–2021, there was an average of 49 child fatalities among those less than five years old. California’s population is nearly 40 million living in 58 counties.^4^ The California Department of Public Health (CDPH) EpiCenter online database reveals that drowning has been the leading cause of injury death among California residents 1–4 years of age for nearly 25 years.^4^ Among children who survive drowning, a large number experience lifelong disabilities that range from minor to severe. The California Department of Developmental Services 2022 Client Development and Evaluation Report documents a caseload of more than 700 persons who require lifelong services as a result of nonfatal drowning.^5^

California is one of four states that address the prevention of residential pool drowning through state legislation. The first California Pool Safety Act went into effect on January 1, 1997, and was updated in 2017. In part, the California Pool Safety Law increased the number of required safety features around residential pools from one to two, expanded allowable safety devices, and expanded the inspection process.^6^ The law covers pools and spas at private, single-family residences at the time of sale, transfer of property, or remodel.

### Purpose of Analysis

The purpose of this data analysis was to examine the annual fatality rates and characterize drowning incidents among California children 1–4 years of age (University of California Irvine IRB #1735.)

## METHODS

We obtained the data for this study from the EpiCenter California Injury Data Online website. This is a comprehensive source of injury data limited to California residents. EpiCenter data includes fatalities, injury hospitalizations, and emergency department (ED) visits.^4^ Drowning fatalities were identified from the CDPH using International Classification of Diseases, 10^th^ Rev, Clinical Modification (ICD-10-CM) cause-of-death codes appearing in the underlying cause-of-death field as follows: W65–W74; X71; X92; Y21; W65–W74; X71; X92; and Y21. We calculated fatality rates from the California Department of Finance Report P-3: Complete State and County Projections Dataset (Baseline 2019 Population Projections; Vintage 2020 Release).^4^

Injury hospitalizations and ED visits were identified from the California Department of Health Care Access and Information (HCAI) Patient Discharge Dataset (PDD) and the ED dataset, respectively. The PDD includes records of inpatient discharges from California-licensed hospitals, including general acute care, acute psychiatric, chemical dependency recovery, and psychiatric health facilities. The ED data includes records of patient face-to-face encounters with clinicians at hospitals licensed to provide emergency medical care. If an ED encounter resulted in a same-hospital admission, the ED encounter would be combined with the PDD record and only appear as a hospitalization. We identified drowning hospitalizations and ED visits using ICD-10-CM codes appearing in any of 25 diagnosis fields and 12 external cause-of- morbidity fields.^7^ On the EpiCenter interactive website, we selected “drowning/submersion” under the Injury Mechanism drop-down menu for deaths, hospitalizations, and ED visits. This included unintentional, intentional, and undetermined intent drownings.

Consistent with California Health and Human Services De-Identification Guidelines, EpiCenter does not provide data for results with fewer than 11 cases. Because of the de-identification guidelines, we were only able to determine that less than 4% of fatal cases were intent unknown or intentional. Less than 1% of hospitalized and ∼0.3% of ED cases were classified as intentional.

We looked at the overall incidence of cases among California children 1–4 years of age for the years 2017–2021 and the rates of fatalities, hospitalizations, and ED visits. Data elements reviewed included age in years, sex of child, race/ethnicity, location of incident, outcome (fatal, hospitalization, ED visit), disposition, and length of stay. Incident location is defined as the body of water, based on ICD-10/ICD-10-CM code descriptions (eg, pool, bathtub, or open body of water). Race/ethnicity is categorized as follows: Hispanic; White; Asian; Black; multiracial; and Pacific Islander. We calculated incidence rates and 95% confidence intervals assuming a Poisson distribution of drowning data. *P*-values were calculated based on the chi-square test by exact calculation method. We used Stata 17.0 SE (StataCorp LLC, College Station TX) for statistical analysis.

## RESULTS

### Incidence

During the study period, 2017–2021, a total of 4,166 drowning incidents were documented. Table 1 shows the annual incidence of drowning by fatality, hospitalization, and ED visit. Table 2 shows the incidence rate (IR) of drowning by age and sex. The incidence of drowning was highest among the 2-year-old age group (*P* < 0.01). Table 3 shows the IR of drowning per month and day of the week during 2017–2021. Drowning was most prevalent in June and July and least prevalent in December and January (*P* < 0.001). Similarly, drowning was more prevalent on weekends compared to weekdays (*P* < 0.001).

**Table 1.** Annual drowning rates of California children 1–4 years of age: 2017–2021.

	Fatalities (N = 234)		Hospitalizations (N = 846)		ED visits (N = 3,086)		Total (N = 4,166)
Year	N	Crude rate^*^	N	Crude rate^*^	N	Crude rate^*^	Population
2017	44	2.2	220	11.1	876	44.1	1,986,642
2018	53	2.4	219	11.1	709	36.0	1,967,438
2019	44	2.2	157	8.2	619	32.2	1,923,620
2020	47	2.4	118	6.3	389	20.9	1,864,194
2021	46	2.5	132	7.2	493	27.1	1,821,672

* Rate: Incidence rate per 100,000 person-year.

*ED*, emergency department.

**Table 2.** Demographics of the drowning incidence of California children 1–4 years of age: 2017–2021.

		Fatalities (N = 234)			Hospitalizations (N = 846)			ED visits (N = 3,086)			Total (N = 4,166)	
		N	%	Rate^*^	N	%	Rate^*^	N	%	Rate^*^	N	Rate^*^
Age (years)	1	84	7.9%	3.7	227	21.2%	9.9	758	70.9%	33.0	1069	46.6
	2	75	5.3%	3.2	283	19.9%	12.0	1061	74.8%	44.9	1419	60.1
	3	48	4.4%	2.0	223	20.4%	9.2	820	75.2%	33.8	1091	45.0
	4	27	4.6%	1.1	113	19.3%	4.6	447	76.1%	18.1	587	23.8
Sex	Male	154	6.5%	3.2	506	21.3%	10.5	1717	72.2%	35.8	2377	49.5
	Female	80	4.5%	1.7	340	19.0%	7.1	1369	76.5%	28.7	1789	37.5

* Rate: Incidence rate per 100,000 person-year.

*ED*, emergency department.

**Table 3.** Timing of the drowning incidence in California children 1–4 years of age: 2017–2021.

		Fatalities (N = 234)		Hospitalizations (N = 846)		ED visits (N = 3,086)		Total (N = 4,166)
		N	%	N	%	N	%	N
Month	1	7.75^*^	9.1%	20	23.6%	57	64.8%	88
	2	13	11.5%	28	24.8%	72	63.7%	113
	3	7.75^*^	5.4%	31	21.6%	105	71.4%	147
	4	24	7.5%	70	22.0%	224	70.4%	318
	5	28	6.8%	97	23.6%	286	69.6%	411
	6	38	4.3%	172	19.7%	664	76.0%	874
	7	43	4.5%	168	17.4%	754	78.1%	965
	8	29	4.9%	118	19.8%	448	75.3%	595
	9	17	5.4%	57	18.0%	242	76.6%	316
	10	7.75^*^	4.6%	39	23.0%	123	71.1%	173
	11	7.75^*^	7.7%	25	24.8%	68	65.4%	104
	12	11	14.7%	21	28.0%	43	57.3%	75
Day of week	Sunday	37	4.2%	183	20.6%	669	75.3%	889
	Monday	24	4.8%	105	21.1%	368	74.0%	497
	Tuesday	34	7.6%	98	21.9%	316	70.5%	448
	Wednesday	30	6.7%	79	17.7%	338	75.6%	447
	Thursday	32	7.4%	87	20.1%	313	72.5%	432
	Friday	38	7.8%	101	20.8%	346	71.3%	485
	Saturday	39	4.0%	193	19.9%	736	76.0%	968

* The number of incidents was recorded as “<11,” and we arbitrarily replaced them with 7.75, contributing to the grand total of 4,166.

*ED*, emergency department.

Drowning incidents among 234 (5.62%) cases were fatal. Of the nonfatal cases, 2,438 (58.52%) patients were treated and released from the ED; 628 (15.07%) required hospitalization for less than a day or overnight; 136 (3.26%) stayed in the hospital for 2–4 days; 82 (1.97%) had hospital stays exceeding four days; and 648 (15.55%) were either transferred from the ED or had unknown dispositions.

### Fatality

The fatality rates varied from 2.2 per 100,000 population in 2017 to 2.5 per 100,000 in 2021, but the observed difference failed to attain statistical significance (*P* = 0.88). This is contrary to the decrease in ED visits and hospitalization during the same period. The case fatality ratio for the 1-year-old age group stood at 7.86% (6.32–9.64%), which was higher than for other age groups (*P* < 0.001). Similarly, the case fatality ratio for males was 6.48% (5.52–7.54%), exceeding the fatality ratio for females, which was 4.47% (3.56–5.53%) (*P* = 0.005). The analysis of fatal drowning incidents among 234 children aged 1–4 years, based on the location, showed that pools were the most common site, accounting for 65% (152 cases). Bathtubs followed at 14% (32 cases), natural bodies of water at 11% (27 cases), and 10% (23 cases) occurred at other or unspecified water sources.

Fatal drowning rates by race/ethnicity were highest among Black and multiracial children (3.3/100,000), followed by White children (3.0/100,000), Hispanic children (2.1/100,000), and the lowest rate was among Asian children (1.8/100,000).

## DISCUSSION

Drowning remains the leading cause of death among California children 1–4 years of age. The annual rate of fatality over the five-year study period did not decline.

### Incidence

Over the five-year study period (2017–2021), 4,166 drowning incidents were reported among California children 1–4 years of age, an annual average of 833 incidents. Of these, nearly 6% were fatal and 94% were nonfatal drownings. The highest IR was among children two years of age. Incidents in the summer months (June and July) and on weekends were most prevalent. Racial/ethnic rates are only available for fatalities due to 2019 modifications to race and ethnicity coding in the HCAI hospital discharge and ED visit data. Fatality rates were highest among Black and multiracial children. The highest rates among Black children have been documented in other studies as well, indicating the need to address disparities.^3^ ^,^ ^8^ ^,^ ^9^ ^,^ ^10^ ^,^ ^11^ ^,^ ^12^

### Fatal

A previous analysis found that annual rates of unintentional fatal drownings from 1999–2020 among California children 1–4 years of age at all locations and at swimming pools did not decline significantly.^13^ For the years 2017–2021, of the 234 fatalities among children 1–4 years of age, 65% occurred at pools, similar to the findings in other studies. The coding does not differentiate the location of pools (eg, residential or community), which are governed by different regulations. However, it is well documented that fatal incidents among young children are most common at residential pools. The National Center for Fatality Review and Prevention (NCFRP) reported that 52% of fatal drownings occurred in a home or residential pool. The Consumer Product Safety Commission Pool or Spa Submersion 2023 Report also revealed that among the fatalities, 84% were at a residential pool.^2^ ^,^ ^3^ ^,^ ^11^ ^,^ ^12^ ^,^ ^14^ This data highlights the need to prioritize prevention efforts that target pool drownings. For nonfatal cases, we could not determine whether the location was a pool or other body of water because of the large proportion of T75.1 ICD-10-CM codes corresponding to “Unspecified effects of drowning and nonfatal submersion.”

### Fatal/Nonfatal

For every fatality among the 1–4-year age group in this study, there were 18 nonfatal incidents. This finding highlights the need to include and compare fatal and nonfatal incidents to better understand the circumstances and modifiable risk factors, such as submersion time before rescue and initiation of cardiopulmonary resuscitation at the site.^15^ ^,^ ^16^ ^,^ ^17^ ^,^ ^18^ The current California pool safety law is SB-442 Public Health: Pools: Drownings 2017.^6^ Based on its provisions, coverage, and inspection requirements, we would not expect to see a reduction in a drowning fatality because 1) fatality is a rare event; 2) we have no documentation of the degree to which pool owners remain compliant after the one-time inspection; and 3) although two “safety features” are required, only one of the options is a best practice — an enclosure that isolates the pool from access to the home meeting California Building Standards Code specifications.

### Addressing Data Gaps

Based on these findings, robust integrated fatal and nonfatal local, state, and national systematic data collection systems that enhance our understanding of the epidemiology and modifiable risk factors including regional differences are necessary to move the needle in decreasing pool fatalities among young children. Comprehensive surveillance systems would also include all portals of data entry, beginning with first responders such as lifeguards, emergency medical services, and law enforcement. Standard definitions and coding using ICD-10 and IC1-10-CM, data source linkages, real-time electronic data entry, timely analysis and reporting, and state-of-the-art technology to improve data variable capture will increase our knowledge and fill data gaps.^19^ ^,^ ^20^ ^,^ ^21^ Preliminary results from the Drowning Death Scene Investigation and the Child Death Review (CDR) Project of the NCFRP also indicate that the widespread use of a standardized tool will fill a significant data gap in knowledge to inform prevention.^22^

Local CDR, a process that allows for a multidisciplinary comprehensive review of child fatalities using data collected from multiple sources, has been used effectively for both surveillance and to inform prevention.^23^ ^,^ ^24^ ^,^ ^25^ The Haddon Matrix model as applied to drowning captures the multiple layers of protection and interventions to interrupt the progression to a death by drowning and can be integrated into case fatality review.^9^ ^,^ ^26^ The Injury Equity Framework theoretic model provides further guidance on systematic data collection and analysis that can be considered in the local child death review process.^27^

In addition to pediatricians (child abuse, intensivists, primary care), local CDR teams ideally include representatives from the county child protective social services, law enforcement, public health and school nursing, and the coroner’s or medical examiner’s offices. The coroner’s office (and often law enforcement) conducts detailed investigations in cases of unexpected child death, especially those who die without being transported to hospitals. Other team members may also have additional interaction with the child or family.^28^ The availability of guidelines on the type of information to gather in fatal drowning cases should improve data capture by CDR team members.

Physician involvement in improving the quality and quantity of data in health record documentation and the subsequent CDR process further details the circumstances and provides insight into future prevention recommendations. Further, reporting to county child protective services if there are concerns for lack of appropriate supervision or safeguards can result in additional resources or services for the family to prevent future incidents. Child death review as a surveillance tool with action recommendations is effective.^23^ ^,^ ^24^ ^,^ ^25^ Pediatricians can advocate for integrating non-fatal drowning into the fatality review.

The American Academy of Pediatrics (AAP) strongly recommends that states establish systematic reporting on the circumstances of drowning.^9^ A robust statewide data collection and analysis system provides information to develop best practices, community interventions, and relevant public policies. The California Legislature found a solution to the statewide drowning data collection gap in reporting fatal and nonfatal incidents when California Senate Bill 855 (Newman, Ch. 817, Stat. 2022: Child Drowning Data Collection Pilot Program) was chaptered into law. Implementation of a data collection system by the CDPH moves us closer to the AAP recommendation that states establish systematic reporting. Strengthening our county CDR teams in reviewing drowning cases and including nonfatal cases for analysis should contribute to a robust statewide surveillance system and can be a model for other states.^9^ ^,^ ^10^ ^,^ ^29^

## LIMITATIONS

There are several limitations to this study. The CDPH EpiCenter online publicly available database, based on vital statistics and hospital/emergency discharge data, enables an analysis of annual rates of drowning incidents only among California residents over time. Children who visit California and our popular tourist attractions are not counted if they sustain a fatal or nonfatal drowning. Moreover, the database is limited in identifying modifiable risk factors and event circumstances that can further inform data-driven prevention strategies and policies.

The ratios of fatal to hospitalization to ED visits may include double counts as some ED encounters may also be included in the hospital data. We do not have exposure data. Additionally, we could not determine the extent of unreported incidents. As Koon pointed out, the CDPH publicly accessible data related to drowning was not designed for research.^14^ Finally, a significant data gap relates to the location of the pool for hospital and ED visits due to ICD-10-CM coding variability.

## CONCLUSION

Drowning remains the leading cause of unintentional, injury-related death among California children 1–4 years of age, as the annual rate of fatality over the five-year study period did not decline. While the EpiCenter California Injury Data Online website is excellent for analyzing annual rates of drowning incidents among California residents over time, it is limited in providing insight into modifiable risk factors and event circumstances that can further inform prevention. Currently, inconsistent and incomplete data hamper the identification and monitoring of trends, risk factors, and prevention recommendations. Incorporating data elements in the context of the injury equity framework will further guide interventions.^27^ Systematic child death review, combined with all child drowning incident investigations can inform evidence-based best practices, community interventions and the implementaton of effective and impactful public policies.
